# Synonymous Constraint Elements Show a Tendency to Encode Intrinsically Disordered Protein Segments

**DOI:** 10.1371/journal.pcbi.1003607

**Published:** 2014-05-08

**Authors:** Mauricio Macossay-Castillo, Simone Kosol, Peter Tompa, Rita Pancsa

**Affiliations:** 1 Vlaams Instituut voor Biotechnologie (VIB) Department of Structural Biology, Vrije Universiteit Brussel, Brussels, Belgium; 2 Institute of Enzymology, Research Centre for Natural Sciences, Hungarian Academy of Sciences, Budapest, Hungary; Indiana University, United States of America

## Abstract

Synonymous constraint elements (SCEs) are protein-coding genomic regions with very low synonymous mutation rates believed to carry additional, overlapping functions. Thousands of such potentially multi-functional elements were recently discovered by analyzing the levels and patterns of evolutionary conservation in human coding exons. These elements provide a good opportunity to improve our understanding of how the redundant nature of the genetic code is exploited in the cell. Our premise is that the protein segments encoded by such elements might better comply with the increased functional demands if they are structurally less constrained (i.e. intrinsically disordered). To test this idea, we investigated the protein segments encoded by SCEs with computational tools to describe the underlying structural properties. In addition to SCEs, we examined the level of disorder, secondary structure, and sequence complexity of protein regions overlapping with experimentally validated splice regulatory sites. We show that multi-functional gene regions translate into protein segments that are significantly enriched in structural disorder and compositional bias, while they are depleted in secondary structure and domain annotations compared to reference segments of similar lengths. This tendency suggests that relaxed protein structural constraints provide an advantage when accommodating multiple overlapping functions in coding regions.

## Author Summary

Certain genomic regions code for multiple, overlapping functionalities that can be detected by analyzing the levels and patterns of their evolutionary conservation. The redundant nature of the genetic code facilitates the appearance of such multi-functional gene regions through evolution. At many of these sites the DNA sequence encodes a protein segment and in parallel to that another function, e.g. regulatory sites involved in translation initiation and transcript splicing. However, it has never been studied how the corresponding protein segments can tolerate that their primary sequences, and consequently their structures, are restricted by the sequences of the overlapping functionalities. To answer this question, we analyzed a recently published, large set of human, potentially multi-functional coding regions for the structural properties of encoded proteins with a variety of computational structure prediction tools. We examined the level of disorder, secondary structure, and sequence complexity of the corresponding protein regions, and found that multi-functional gene regions translate into protein segments that are significantly enriched in structurally disordered and compositionally biased regions, while they are depleted in secondary structure and domain annotations compared to reference segments of similar lengths. This tendency suggests that protein structural disorder provides evolutionary advantage when accommodating multiple overlapping functions in coding regions.

## Introduction

The rapid development of comparative genomics approaches along with the fast increase in the number of sequenced genomes provides us with a clearer view on evolutionarily conserved and hence potentially functional genome regions [1]. All the underlying approaches are based on the notion that mutations affecting functionally important regions are deleterious and hence are likely to be eliminated from the population by purifying selection, while mutations affecting ‘junk DNA’ are neutral and can accumulate during evolution [2], [3]. The increase in the number of sequenced genomes supports the discovery of new functional elements of different types [4]–[8] by enhancing the power and resolution of detecting constrained regions [9]–[13]. However, these depend not only on the number but also on the diversity of species compared [14]. Sequences from closely related organisms might simply not have had enough time to change and hence they can be mistakenly assigned as conserved without functional importance. On the other hand, alignments from distantly related genomes are suitable for detecting constrained genome regions, although they may overlook recently evolved functionalities.

**Figure 1:**
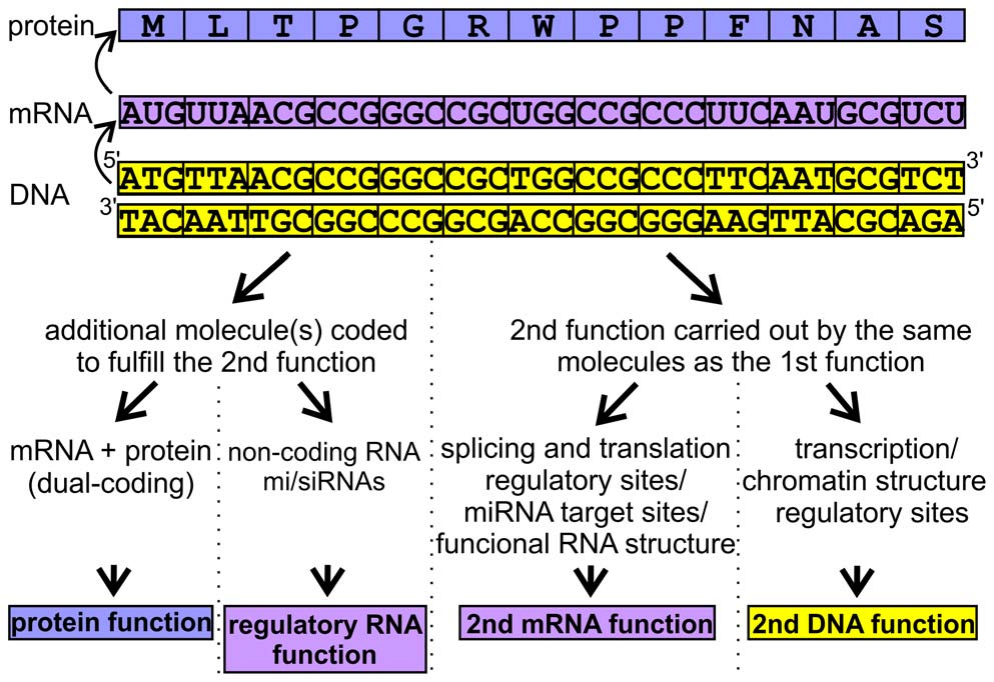
Possible overlapping functions fulfilled by synonymously constrained coding regions. The primary function of a given coding DNA segment is indicated at the top, while at the bottom, different types of possible second functions that could be maintained by the same DNA segment, are summarized. These functions can be grouped into two major classes, depending on the need for an extra molecule fulfilling the extra task. These two are then further divided according to the molecule type involved.

A recent comparative genomics study of 29 mammalian species identified constrained elements that cover 4.2% of the human genome [15]. Since 20 of the 29 genomes were selected and newly sequenced for the analysis, the power of detecting evolutionary constraints was largely improved compared to previous approaches [16], [17]. In the resulting robust genomic alignment, the levels and patterns of evolutionary conservation reflected the number and type of different functionalities fulfilled by a given genome region. Lindblad-Toh and colleagues applied phylogenetic codon models to find small windows in known open reading frames (ORFs) that exhibited unusually low rates of synonymous substitution and identified a surprisingly large number (∼10000) of synonymous constraint elements (SCEs) within human coding exons [15]. SCEs are protein-coding regions in which synonymous mutation rates are extremely low compared to the average rate of the complete ORF in which they are located, as well as compared to the average rate of the given ORFome, indicating additional sequence constraints beyond those dictated by the structure and function of the protein [15], [18]. These additional constraints most frequently stem from the demands of regulatory sites involved in translation initiation and transcript splicing. SCEs can also contain target sequences for miRNAs, or sequence-specific DNA-binding proteins such as transcription factors. They could code for another protein segment in shifted reading frames (dual-coding), for functionally important mRNA structures, or for non-coding/regulatory RNAs (Figure 1). Most of these potential overlapping functions are present among the examples described by Lin MF and colleagues [18]. In these cases, one can imagine that the DNA sequence bears signs of competing demands of 1) DNA function, 2) RNA function, and 3) protein function. The interplay between diverse functionalities could in principle result in DNA regions serving three or even more different needs. Interestingly, in these dual- or multi-functional DNA regions the changes in the amino acid sequence of the encoded protein segments are restricted by the second functionality, which could influence their capability to form structured elements and folded domains. The question is how the corresponding protein segments can overcome these difficulties. In our opinion, such protein regions might show rather relaxed structural constraints, i.e. enrichment in low sequence complexity or, at least, in structural disorder.

Structural disorder in proteomes was discovered only a decade ago [19]–[22], and since then intrinsically disordered proteins (IDPs) constitute a new, fast developing field of structural biology [23]. Intrinsically disordered regions (IDRs), which function as ensembles of different conformations [22], are well predictable based on their unique amino acid composition [20], [24], widespread in eukaryotic proteomes [25]–[27], and are abundant in proteins of signaling and regulatory roles [19], [28]. From an evolutionary point of view, due to their increased tolerance against mutations, IDRs undergo more rapid changes than globular domains [27]. Additionally, they are frequently subject to enhanced positive Darwinian selection [29]. Although proteins containing long IDRs are evolutionarily more constrained, IDRs themselves are less constrained and more enriched in single nucleotide polymorphisms (SNPs) than any regular secondary structure type [30]. The increased tolerance to mutations stems from the lack of defined secondary or tertiary structure and consequently reduced structural constraints, which predisposes IDRs to be more tolerant of restrictions affecting their coding sequences. For instance, multifunctionality in alternatively spliced gene regions that can give rise to distinct protein chains in different reading frames appears to correlate with protein disorder [31].

Here, we describe a comprehensive computational analysis of the structural preferences of protein segments encoded by potentially multi-functional gene regions. This work aims to provide a better understanding of the limitations of the genetic code in terms of encoded complexity through the detailed analysis of genomic sites that take advantage of its redundant nature.

## Materials and Methods

### SCE data collection

Data on SCEs detected at three different resolutions have been downloaded from the webpage published in support of the 29 mammalian genomes project [15], [18]. The provided genomic locations apply for the NCBI36/hg18 assembly of the human genome. The analysis was performed on all three datasets of 9, 15 and 30 codon resolutions (containing 11882, 10757 and 8933 SCEs, respectively).

### Identification of SCE-encoded protein segments

We have used the programmatic access option of the Ensembl database [32] release 54 to find the protein segments corresponding to the listed SCE genomic locations. The exon segment(s) listed for each SCE were mapped onto the protein coding sequences (CDSs) of all transcripts of the given gene. A match against the canonical isoform's CDS had preference over the others and only one segment was accepted even in case of multiple matching transcripts. The boundaries of the match with the CDS explicitly defined the SCE-encoded protein regions; all residues with at least one nucleotide overlapping the SCE sequence were taken into account. In the majority of the cases the mapping could be performed directly, however, in some cases we had to use the Basic Local Alignment Search Tool (BLAST; version 2.2.25) to locate the SCEs on the CDSs due to nucleotide mismatches. In these BLAST searches, soft masking and the dust option were switched off. In the case of segments coming from the 9- and 15-codon resolution datasets, the short option of nucleotide BLAST was applied.

For a small fraction of the data (135 (∼1.14%), 120 (∼1.12%) and 8 (∼0.1%) data points for the 9, 15 and 30-codon resolution datasets, respectively) no reliable match between the SCE sequence and the corresponding CDSs was obtained due to multiple consecutive gaps. These cases were excluded from further analyses.

### Prediction of protein structural properties

The IUPred method (long window option) [33] was used to predict protein disorder, while regions of low sequence complexity were defined by SEG (default parameters) [34], [35]. Secondary structure was assigned by PSIPRED v3.3 (without using PSI-BLAST profiles) [36], and domains were identified by the PfamScan [37] tool using only the more reliable A-class domains/motifs/repeats/families listed in Pfam release 25.

Here, we briefly summarize the considerations that influenced our choice of prediction methods for this study. IUPred is a widely used disorder prediction method, one of the few that are freely available not only as a web server but also as a ready to install software package. It is fast and can smartly handle obstacles, such as letter codes of non-conventional amino acids or extremely long proteins, making it suitable for analyzing proteome-scale data. Most importantly, it is based on clear physical principles that allow for easy understanding and interpretation of its prediction results [33]. IUPred is thought to provide direct proof for the existence of structural disorder, relying purely on residue-residue interaction energies, without being pre-trained on protein disorder datasets [33], [38]. Additionally, IUPred is considered to be rather conservative, which means that it is not prone to overestimate the abundance of structural disorder. Another feature that makes IUPred an ideal choice for the current study is that, in contrast to many other predictors, it does not take sequence complexity into account when estimating the disordered nature of a protein region, i.e. it is orthogonal to SEG. SEG is a widely used method for the identification of low sequence complexity segments, even applied as a pre-filtering step in BLAST searches [39]. SEG is based on a simple formula that describes the compositional complexity of a given sequence window with defined length and assigns it as low complexity if the calculated value is below a given cut-off [34], [35]. Due to this, the prediction outputs provided by SEG are also easy to understand and interpret. Finally, PSIPRED is a popular secondary structure prediction method that is reasonably accurate and fast, and besides the web server, it also has a freely available version for local use. PSIPRED can be optionally run without creating PSI-BLAST profiles that enables predictions on proteome-scale data in reasonably short times.

The structural properties of each protein segment (SCE-encoded or reference) were obtained by retrieving the corresponding values from the predictions of the full-length proteins. This way, the segments were studied in their natural sequence environment and systematic termini biases could be avoided. The following measures were used to describe the structural properties of the segments: 1) the fraction of disordered residues (scoring ≥0.5 by IUPred), also referred to as disorder content, 2) the fraction of residues in low-complexity regions, 3) the fraction of residues in regular secondary structure elements (helix or extended), also referred to as secondary structure content, and 4) the fraction of residues in any predicted Pfam entities. The segments were also grouped in a binary manner for each predicted structural property (e.g. disordered/non-disordered); we assigned a segment to a given structural property if at least 50% of its residues were predicted as such.

Since PSIPRED failed to predict secondary structure for proteins larger than 10,000 residues, we had to exclude the *titin* gene [Ensembl 54: ENSG00000155657] and its products from our analysis to maintain the consistency of our data. Due to this reason, there were 13, 9 and 6 SCEs excluded from the 9, 15 and 30-codon resolution SCE datasets, respectively.

### Selection of suitable reference datasets

For each SCE-encoded protein segment, a segment of equal length was randomly picked from the SCE-containing subset of human proteins in a way that the residue boundaries of the proteins were not exceeded. This way undesired reference segments containing artificially fused termini of two different proteins could be avoided. Consequently, we acquired one set of randomly selected reference segments for each SCE dataset and defined their structural properties as previously described.

### Identification of experimentally validated disordered protein segments overlapping SCEs

We looked for direct matches between the 15-codon resolution SCE-encoded sequences and the human DisProt 6.02 proteins [40], and counted the overlaps with the annotated disordered regions. To gain suitable reference values, we randomly selected, for each human DisProt protein, one human canonical protein matching in length (within +5%), and transferred the annotated disordered segment boundaries onto them. Then we matched the SCE-encoded sequences against the randomly selected proteins and counted the overlaps with the segment boundaries. This way, we could maintain the length distribution and the fraction of N- or C-terminal segments of the DisProt set within our random set. The whole procedure was repeated five times to gain multiple reference values. Then we used Yates' chi-square test to compare the median of the reference values (expected) to the tested value.

### Investigation of experimentally validated splicing factor binding sites (SFBSs)

We downloaded a set of 211 experimentally verified SFBSs from the SpliceAid-F database [41] that reside in human exons and span more than 4 but less than 50 nucleotides. After filtering out mutant genes and sites with redundant chromosomal locations, we obtained 64 unique binding sites. Out of these, 62 could be successfully mapped onto Ensembl transcripts. The two SFBSs in the FAS gene overlapped with several transcripts coding for two distinct protein chains (due to two overlapping exons in shifted reading frames). Here, we accepted two transcripts per SFBS representing distinct coding frames. The corresponding protein segments were identified for all SFBSs, similarly as for SCEs, and their structural properties were calculated in the same way. The random selection of reference segments of equal length was also performed for SFBSs using the whole human canonical proteome.

### Statistical evaluation

Since our datasets failed the Kolmogorov-Smirnov normality test, we applied Mann-Whitney U test to compare the properties of SCE-containing proteins (represented by the 9-codon resolution dataset, the one with the highest number of entries) with the human canonical proteome, and also to compare the four predicted structural properties of SCE-encoded and SFBS-encoded protein segments to those of their equivalent reference segments. Due to the multiplicity of comparisons between these datasets, Bonferroni corrections were applied, which resulted in lowered significance thresholds of p = 0.01 and p = 0.0125, respectively.

We also applied Yates' chi-square test to compare the SCE-encoded segment datasets (observed values) with their reference sets (expected values) using the number of at least 50% assigned segments in case of each structural property. Again, Bonferroni correction was applied when setting the significance thresholds (p = 0.0125).

For testing the correlation between the structural properties of the SCE-encoded segments and the detection window size, we applied Spearman's correlation test. To show that the structural properties of the three SCE datasets differ we used Kruskal-Wallis tests. The differences were further analyzed by Dunn's multiple comparison tests.

**Figure 2:**
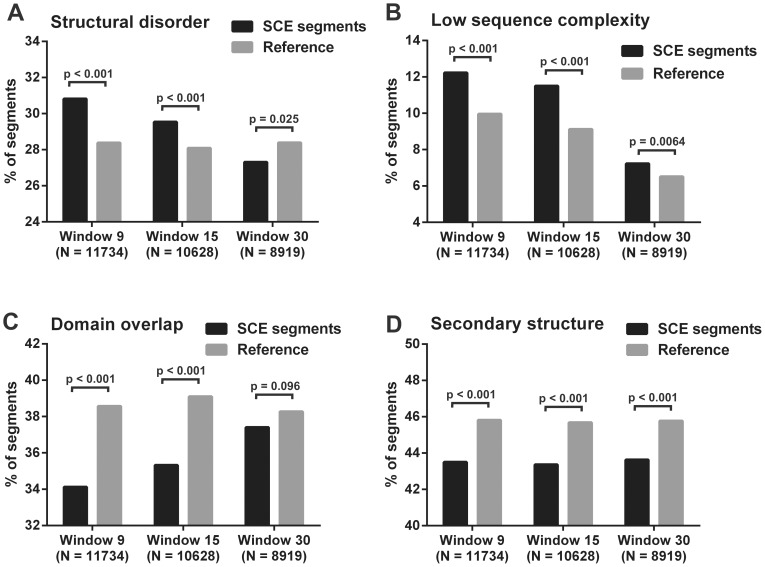
Comparison of SCE-encoded protein segments with reference segments from four structural aspects. Human SCE-encoded protein segments are compared to randomly selected protein segments of the SCE-containing proteins with same length distribution from four structural aspects. A segment is accepted to belong to a given structural property if at least 50% of its residues are positively assigned by the corresponding prediction method. Percent of segments assigned with A) structural disorder (IUPred), B) low sequence complexity (SEG), C) domain annotation (PfamScan) and D) secondary structure (PSIPRED) for SCE-encoded and reference segment datasets of all three detection resolutions. The numbers of segments for each property were compared between the SCE and reference datasets using Yates' chi-square test with the corresponding p-values indicated above the bars.

In the case of the SFBS data, the residue-level analysis was performed by Yates' chi-square test. We counted the overall number of residues of predicted disorder/low complexity/domain or secondary structure in SFBS segments and used these (and their complementary values) as observed values for testing. The expected values were obtained by multiplying the sum of SFBS segment lengths by the fraction of residues assigned to the given structural property in the whole canonical proteome. Bonferroni corrections were applied on the significance thresholds. GraphPad Prism 6 was used for statistical testing and preparation of Figure 2.

### Description of proteins used as case studies

We have used the following Ensemble proteins as examples: HOXA2 [ENSEMBL 54: ENSP00000222718]; canonical BRCA1 [ENSEMBL 54: ENSP00000350283]; shorter BRCA1 isoform translated from the introduced alternative translation start site: [ENSEMBL 54: ENSP00000377288]; canonical FAS [ENSEMBL 54: ENSP00000360942], non-canonical FAS in alternative reading frame: [ENSEMBL 54: ENSP00000318464]; CBP [ENSEMBL 54: ENSP00000371502], and p300 [ENSEMBL 54: ENSP00000263253].

## Results

### Structural analysis of human SCE-encoded protein segments

The large collection of human SCEs (three datasets representing detection resolutions of 9, 15 and 30 codons) was adopted from Lin et al [18]. Due to the stringent filtering criteria applied in detecting SCEs, we consider the published dataset as a collection of potentially multi-functional coding regions, and hence did not perform any further filtering steps. The SCEs were mapped onto human proteins and the structural properties of the resulting protein segments (11734, 10628 and 8919 segments in the three datasets, respectively) were determined by a variety of structure prediction methods. IUPred was used to predict structural disorder, SEG for low sequence complexity, and PSIPRED v3.3 for secondary structure. Pfam entities (domains/families/motifs and repeats; hereafter collectively referred to as domains) were identified by the PfamScan method. The predictions were always obtained for full-length proteins and the segments of interest were excised subsequently. The fractions of assigned residues were defined in each SCE-encoded protein segment for each structural property. All details on SCE-encoded protein segments are provided in the Supporting Information, Tables S1, S2 and S3 for resolutions 9, 15 and 30 codons, respectively.

First, we compared the set of SCE-containing proteins with the whole human canonical proteome to identify an adequate reference dataset for statistical comparisons of the SCE-encoded protein segments. SCE-containing proteins were significantly longer, more disordered and enriched in low complexity regions compared to the proteome (Figure S1). Consequently, we used only this subset of proteins for obtaining reference segments, to ensure that the observed structural differences do not derive from the general distinctive characteristics of proteins containing SCE regions. The reference segments were randomly selected from SCE-containing proteins, preserving the length-distribution of the SCE-encoded segments, and their structural properties were determined as described above. Information on the randomly selected datasets is provided in Tables S4, S5 and S6 for resolutions 9, 15 and 30 codons, respectively.

The number of segments with 50% or more residues assigned to a given structural property was compared between the SCE-encoded segments and the reference segments (Figure 2). Statistical comparisons showed that significantly more SCE-encoded protein segments of high resolution (9 and 15 codons) are structurally disordered and compositionally biased than reference segments, while there are less SCE-encoded protein segments assigned with secondary structure and annotated domain regions (Yates' chi-square tests). In case of the low, 30-codon resolution, however, the differences between the numbers of SCE and reference segments calculated for relative domain overlaps and structural disorder were below the threshold of statistical significance (Figure 2A, 2C).

The fractions of residues positively assigned with the structural properties were also directly compared between the SCE-encoded and reference segments using Mann-Whitney U test (Figure S2). The results of this approach were in agreement with the results described above and confirmed the previous analysis. Data on these statistical analyses are provided in Tables S7 and S8.

### The structural properties of SCE-encoded protein segments correlate with the window size of SCE detection

Three different datasets were obtained by varying the window size when screening the genome for SCEs [18]. The above described differences between the structural properties of the SCE and reference datasets seemingly increased with decreasing window size, i.e. increasing resolution (Figure 2). To further investigate this relationship, we applied different statistical approaches. First, we made an attempt to choose one descriptive value for each structural property that represents the distribution of the data well, which was then correlated with the window size used for SCE detection. We used the third quartile (75th percentile) for structural disorder, the median for secondary structure, and the 90th percentile for low sequence complexity. Because of their binary nature, data on domain content could not be represented by a single value and hence were not correlated with the window size (small segments are located either within or outside of domains but rarely at the borderlines). Spearman's correlation showed that each structural property can be described as a monotonic function of the detection window size. Disorder and low sequence complexity content increased with decreasing window size and therefore gave a negative Spearman's rank correlation coefficient (r = −1 in both), while the correlation between secondary structure content and window size was positive (r = 1).

These values demonstrate the monotonic relationship between the structural properties and window size, but, since there are only three data points corresponding to the three windows applied, we additionally used a direct approach to analyze the differences between the whole datasets. For each investigated structural property, we applied Kruskal-Wallis test to see whether the three datasets significantly differ. Dunn's multiple comparison tests were used to further study these differences and they showed that each dataset significantly differs from the other two, considering both low sequence complexity and secondary structure content. The 9- and 15-codon resolution datasets did not differ significantly in disorder and domain residue content. However, the 9- and 30-codon resolution datasets showed significant difference in all four properties (Table S9). Since the reference datasets showed only negligible differences in their structural properties (Figure 2, Figure S2), we can assume that the above described significant structural differences stem from the multi-functional nature of SCEs and are not due to the difference in their length distributions.

This correlation between window size and detected structural properties is expected due to the short length of regulatory elements. In fact, most regulatory elements on both, the DNA- (e.g. transcription regulatory sites) or RNA- (e.g. translation initiation and splicing regulatory sites) levels are usually less than 15 nucleotides in length, which is shorter than the window size of the highest resolution detection. This means that at larger window sizes the actual SCE covers only a small fraction of the window (15 and 30 codons), i.e. the measured structural property represents a mixture of synonymously constrained and single constrained regions, making it difficult to sort them out. Considering this, the gradual diminution of structural bias with window size supports our original assumption that protein-coding sequences under selection for overlapping functions are subject to locally reduced structural constraints. The presence of this relationship in our datasets also provides good support for our approach and indicates its specific nature.

### Experimentally validated disordered protein segments are significantly enriched in SCEs

We directly matched the 15-codon resolution SCE-encoded protein sequences onto human proteins in DisProt 6.02, the database of experimentally verified disordered protein segments. We found 67 matches that completely (40) or partially (27) overlapped with the annotated disordered segments. Applying equivalent random reference sets (see Methods) instead of DisProt, we obtained 23, 29, 40, 41 and 43 matches (median = 40), i.e., DisProt regions contain significantly more SCEs than expected by chance (Yates' chi-square test, p = 2.693E-05).

### Analysing the structural properties of protein segments overlapping experimentally identified splicing factor binding sites (SFBSs)

The SCE datasets used for the above statistical analyses were generated by the *in silico* detection of low synonymous mutation rates in the genome which resulted in a large amount of data providing suitable statistical power for the analyses. Unfortunately, the size of the datasets did not allow for individual observations and experimental verification of the potential secondary functionalities in the identified regions. Therefore, a smaller set of experimentally validated exonic SFBSs was used to probe into the structural properties of protein segments overlapping with multi-functional coding regions.

The frequent occurrence of splicing regulatory sites in the coding regions of human mRNAs is one of the most prominent factors contributing to the large number of detected SCEs [18], which makes them ideal candidates for a more detailed analysis. A set of human exonic SFBSs verified in binding and splicing assays has been downloaded from the SpliceAid-F database and filtered for length and redundancy. Finally, 64 single-nucleotide resolution binding regions were mapped onto protein-coding Ensembl transcripts (out of these, only three overlap with SCEs, so we can consider these datasets as independent) and the corresponding protein segments were subjected to similar structural analyses as previously described for SCEs (details are provided in Table S10).

The comparison of the structural properties of SFBS-overlapping protein segments with the corresponding reference set showed enrichment in structural disorder and depletion in predicted secondary structure. Due to the considerably smaller size of this dataset, the observed tendencies are not as pronounced as in the case of SCEs, but they are still statistically significant (Mann-Whitney U tests; p-value = 0.003 and p-value = 0.011 for disorder enrichment and secondary structure depletion, respectively). In domain annotations and low sequence complexity content, on the other hand, they showed no significant difference from the reference (Mann-Whitney U tests; p-value = 0.090 and p-value = 0.422, respectively). A general bias in experimental data towards domain regions of proteins is probably the cause of the slight enrichment of SFBS-overlapping protein regions in domain residues. This “domain-bias” in experimental research is possibly the result of the preferential investigation of mRNAs which carry mutations potentially causing splicing defects that affect the functionality of the encoded protein and thus cause disease. The SpliceAid-F database is rich in data derived from splicing assays carried out with mutated genes, supporting this explanation. In contrast to disordered regions, functional domains are more sensitive to alterations, including splicing defects as well as single-residue changes. This is due to the fact that the functionality of domains depends usually strictly on structure and the change of one critical residue can result in the loss of structural stability and impair function. IDRs, on the other hand, are more robust and, because their functionally important residues are only located in short stretches (short linear motifs), less affected by missense mutations. In accord, we can assume that in our SFBS set the relatively frequent overlaps with domain annotations are caused by the bias in selecting mRNA segments for experiments, and not by the biased nature of SFBS in general.

The SFBS-encoded protein regions did not differ significantly from the random set in their low sequence complexity content (Mann-Whitney U test; p-value = 0.422), which is again probably due to the domain bias and the fact that the default window size of the SEG algorithm is 12 residues, more than twice the size of protein fragments overlapping with SFBSs (mean = 5.5 residues, median 4 = residues). This latter problem, unfortunately, cannot be overcome by substantially decreasing the window size of SEG for our purpose, because it would compromise the reliability of the method.

We additionally compared the structural properties of the SFBS-encoded protein segments and the human proteome at the single-residue level. The SFBS-overlapping protein residues show an almost two-fold enrichment in structural disorder (Yates' chi-square test; chi-square = 114.161; p = 0) and an approximately 1.5-fold enrichment in low sequence complexity (chi-square = 10.909; p = 9.6E-04). Also, they display a strong depletion in secondary structure of approximately 2/3 (chi-square = 32.1; p = 1.0E-08) and 1.5-fold enrichment in residues with domain annotations (chi-square = 66.613; p = 0).

### Demonstrating the structural properties of SCE-encoded protein segments with specific examples

The second functionality of SCEs - besides protein coding - is defined in only a few cases. Here, we list some well-known examples of different types of overlapping functionalities together with their protein structural properties.

**Figure 3:**
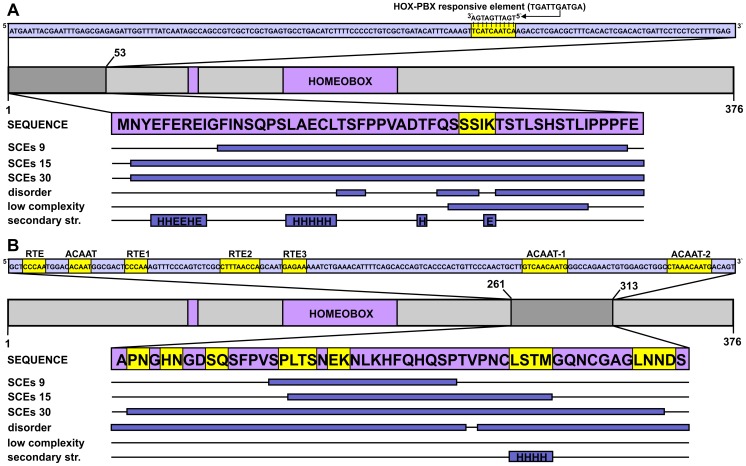
DNA-level secondary functions in coding regions: The case of the *HOXA2* gene. The homeobox protein Hox-A2 is represented by a light grey bar, with its sole domain (homeobox) and antp-type motif colored purple (residue boundaries assigned based on the UniProtKB annotation) and its SCE-overlapping N-terminal region marked by dark grey. The CDS corresponding to this segment is shown above the domain map in a light blue box with the region of multi-functionality (a HOX-PBX responsive element) highlighted in yellow. The corresponding peptide sequence is presented in a purple box with the precise locations of detected SCEs, predicted disordered regions, low sequence complexity segments and secondary structure elements (H – helix, E – extended) represented as dark blue bars below the protein sequence. B) The enhancer-rich region corresponding to residues 261–313 of the same Hox protein is presented in a similar fashion as in panel A.

The *Hox* genes are rich in SCEs, primarily because of the large number of expression regulatory elements embedded in their coding exons [18]. In *HOXA2*, protein coding overlaps with two distinct synonymously constrained regions (both detected at all three resolution levels), each covering previously described enhancer elements that act in distinct regions of the developing brain (Figure 3). In overlap with residues 35–38, a highly conserved HOX-PBX responsive element was reported to drive expression in rhombomere 4 [42]. More downstream (in the range of residues 261–313), a series of SOX2 binding sites was shown to drive expression in rhombomere 2 [43]. Both regions are located outside the sole domain of the protein (the homeobox) and a pronounced shift towards lower structural constraints was detected in both. The residues overlapping the HOX-PBX responsive element were predicted as having low sequence complexity and partial disorder (Figure 3A), and the corresponding region was devoid of regular secondary structure elements. The two residues predicted to fall in helical and extended conformation in this region cannot form a real secondary structure and also, their corresponding PSIPRED confidence scores were very low, so we considered them erroneously assigned by the prediction method. The other segment of 53 residues, although not displaying reduced levels of sequence complexity, was predicted to be almost completely disordered and contained a single, four-residue predicted α-helix with relatively low confidence values (Figure 3B).

**Figure 4:**
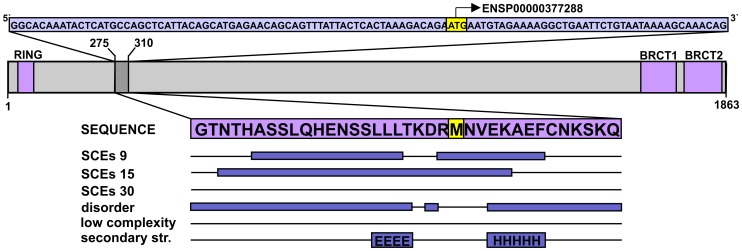
The alternative translation start site within BRCA1 translates into a mostly disordered protein segment. The CDS fragment corresponding to residues 275–310 in the canonical BRCA1 isoform is presented in a light blue box at the top, with a validated alternative translation start site (ATSS) highlighted in yellow. The domain map of the canonical isoform is shown below the CDS with the domains coloured purple (residue boundaries assigned based on the UniProtKB) and the region surrounding the mentioned ATSS marked by darker grey. The protein segment in question is enlarged from the domain map and the identified SCEs and predicted structural properties are indicated below by dark blue bars, as explained for Figure 3.

Breast cancer type 1 susceptibility protein (BRCA1) presents a good example for SCEs overlapping with validated translation initiation regulatory sites. BRCA1 is an E3 ubiquitin ligase playing a central role in DNA damage response [44], [45]. The protein contains an N-terminal RING (really interesting new gene) domain and two tandem BRCT (after the C-terminal domain of a breast cancer susceptibility protein) domains in its C-terminus. These are separated by a more than 1500 residues long, experimentally validated disordered region [46] that mediates a plethora of interactions. Only one synonymously constrained region was detected in *BRCA1*, and it overlapped with an alternative translation start site, which was shown to mediate the translation of a shorter BRCA1 isoform that lacks the RING domain. The corresponding segment of the canonical protein is in the long linker region, and is predicted to be mostly disordered with only a few predicted secondary structure elements (Figure 4). Nuclear magnetic resonance (NMR) and circular dichroism (CD) spectroscopy experiments performed on larger segments containing this region confirmed that this part of BRCA1 is disordered and forms only very limited amounts of secondary structure, if any [46].

**Figure 5:**
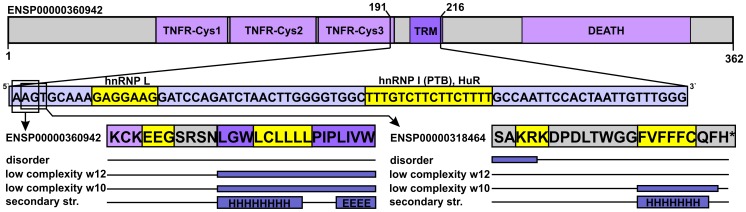
Validated splicing factor binding sites embedded in a dual-coding region. The domain map of the canonical apoptosis-mediating surface antigen FAS is shown at the top, with domains marked by light purple and the only transmembrane region (TRM) marked by darker purple (residue boundaries assigned based on the UniProtKB). The boundaries of the region that overlaps the two splicing factor binding sites are provided, and the CDS corresponding to the given region is presented below the domain map. The two splicing factor binding sites are highlighted by yellow in the CDS with the names of the corresponding splicing factors indicated. The overlapping residues are similarly highlighted in the sequences of the two distinct protein isoforms. The predicted protein structural properties are indicated below as in Figure 3.

The *FAS* gene has a relatively long region annotated as dual-coding (i.e. overlapping exons translated into protein sequence in different reading frames) that contains two adjacent, experimentally validated splicing factor binding sites. In fact, these two sites represent triple-function regions and serve as examples for sites involved in splicing regulation. To the best of our knowledge, such sites have not been described in eukaryotic genomes before. We examined the corresponding regions of both isoforms from a structural aspect (Figure 5). In the canonical protein chain, the three residues corresponding to the shorter binding site do not appear to be disordered or of low complexity, and secondary structure elements are also not predicted here. Interestingly, the residues overlapping the longer binding site reside within the only transmembrane helix region of the protein (assigned by UniProtKB [47]). This region is predicted as low complexity by SEG, since five out of six residues are leucines with only a single cysteine breaking the repeat. Obviously, it is not predicted as disordered by IUPred, which assigns very low scores to a stretch of hydrophobic residues (meaning highly ordered). In the other isoform, the residues overlapping with the binding sites are located in the C-terminal tail and they are not assigned as membrane spanning. The residues corresponding to the shorter binding site are at the border of a predicted disordered region, while the longer binding site encodes for residues predicted to be of low sequence complexity when using SEG with window size 10.

It is clear that in both cases the triple functionality is somewhat compensated for by the protein. The shorter site is located in a disordered region in at least one of the two protein chains, while the long binding site encodes for compositionally biased segments in both. Interestingly, Lin *et al* did not detect SCEs overlapping this region by any of the three applied resolutions. However, considering their rather stringent filtering criteria [18], this could also have other reasons than the lack of low levels of synonymous rate constraint.

**Figure 6:**
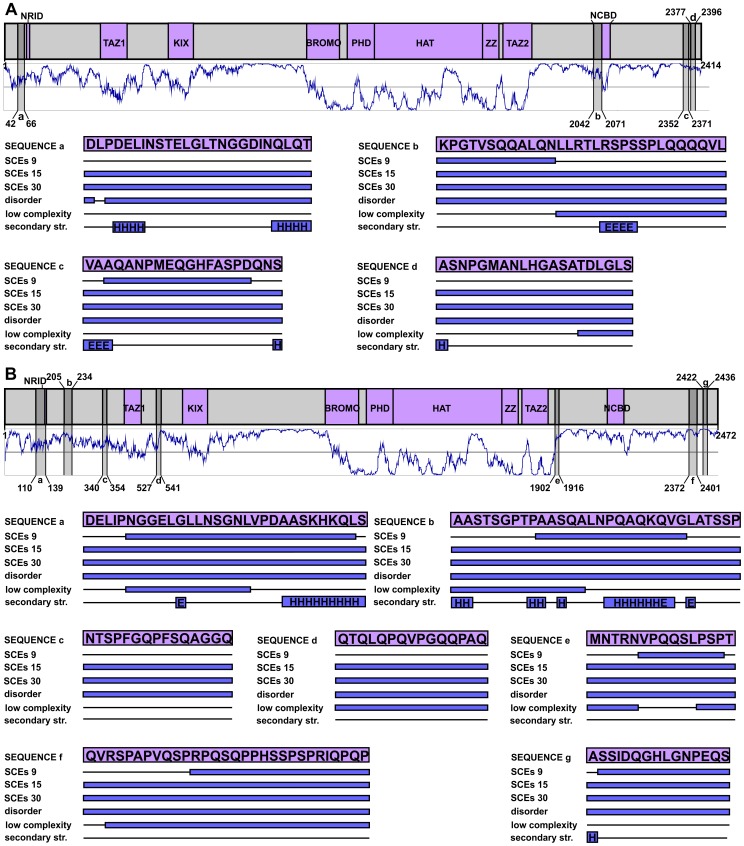
The SCEs of human CBP and p300 are differently distributed along their chains and avoid structured domains. The human p300 (A) and CBP (B) are represented by grey bars at the top part of the panels, with their domains (boundaries adopted from a relevant review [19]) coloured purple and their regions corresponding to 15-codon resolution SCEs coloured with darker grey. Below the domain maps the predicted IUPred disorder patterns are shown in dark blue, where values above 0.5 are interpreted as disorder. The SCE-encoded regions are lettered from the N- towards the C-terminus in each protein and are reflected onto the prediction curves. Their structural properties are provided as in Figure 3.

At the 15-codon resolution, Lin and co-workers detected SCEs in about 6000 human genes (∼35% of the genes). This vast set of genes was checked for biases towards certain Gene Ontology (GO) annotations, and genes involved in “chromatin modification” turned out to be the most enriched in SCEs (∼twofold enrichment) [18]. For instance, several SCEs were detected in the genes of the modular transcription coactivators CBP (CREB-binding protein) and p300 (E1A binding protein p300), and we therefore investigated the structural properties of the corresponding protein segments (Figure 6). Interestingly, despite CBP having seven, and p300 having four, SCE-overlapping regions (in the dataset of 15-codon resolution), none of these is located in well-folded domains. In p300, one of the four SCEs overlaps with the nuclear coactivator binding domain (NCBD), which was previously described as a molten globule [48] and was predicted to be completely disordered (Figure 6A). In CBP, one of the seven SCEs overlaps with the nuclear receptor interacting domain (NRID), which actually is a short binding motif that lies in a segment predicted to be completely disordered in both proteins (Figure 6B). In fact, all 11 regions are predicted to be completely disordered by IUPred and 7 overlap with regions of predicted low sequence complexity. At the same time, none of them show more than 50% secondary structure content, and six of them have no secondary structure elements (or contained only a single residue assigned as such). Interestingly, despite the high sequence similarity of the two proteins, the SCE-overlapping regions are differently distributed along their chains. This could have two reasons: 1) the differences between the structure of the two genes demand different splicing regulation (for instance, CBP has much longer introns resulting in a large difference in overall gene sizes) or 2) the overlapping functionalities evolved after the divergence of CBP and p300 from the ancestral gene.

## Discussion

It has been previously demonstrated that evolutionary rates of proteins are constrained by additional functions encoded by their genes, for instance, when functional RNAs are encoded on the same or opposite strand as protein chains [49]. Apart from a specific study on human dual-coding regions [31], however, such multi-functional coding regions have never been comprehensively studied from the protein structural aspect. We performed a thorough computational analysis on such regions using several carefully chosen structure prediction methods.

First, we investigated this phenomenon on a large scale, by mapping a recently published, high-resolution dataset of human SCEs onto the human proteome and predicting the structural features of the resulting protein segments. We observed a significant enrichment of structural disorder and low sequence complexity, and depletion in regular secondary structure elements and domain annotations. These results imply that the increased functional demands on coding regions of the DNA coincide with structurally biased segments on the protein level. In general, structurally disordered and/or compositionally biased protein segments have lower structural constraints than regions of regular secondary structure elements or globular domains, which confers them enhanced mutation tolerance [27]. Due to this reason, such structurally less constrained protein regions can obviously better accommodate restraints affecting their coding regions. Here, we provide evidence that the encoded polypeptide is compositionally and/or structurally biased in diverse cases of multiple coding, including a variety of short regulatory elements (either on DNA- or on RNA-level).

The observed structural effects were more pronounced when the actual regions of multi-functionality were identified with higher precision. We observed a decrease in relative structural disorder and low sequence complexity content of SCE-encoded segments with increasing SCE detection window size, while their secondary structure content was positively correlated. Also, the datasets of different resolutions were found to be significantly different from each other for most of the structural properties investigated. Larger windows also span protein segments devoid of overlapping functionalities. Hence, the gradual decrease of structural deviations with increasing window size can be considered as further evidence for counterbalancing forces that maintain weak structural constraints in the affected protein regions selectively.

As another proof of the structurally biased nature of protein segments encoded by multi-functional gene regions, we found that the validated disordered regions of the DisProt database are highly enriched in SCEs compared to equivalent reference segments.

To provide further evidence for our hypothesis, we also analysed an independent set of human exonic splicing factor binding sites that have been experimentally validated and identified at single nucleotide resolution. Despite the paucity of data and the observed bias towards domain regions, we found a significant enrichment in structural disorder, depletion in secondary structure elements, and, on a residue-level, enrichment in low sequence complexity in these segments. These results obtained at single nucleotide resolution further strengthen our initial hypothesis about a compensatory mechanism playing a role in multi-functional coding regions. It seems that multiple demands on DNA level coincide with a local decrease of protein structural constraints even within the boundaries of domains. Additionally, the surprisingly small overlap between the computationally detected SCEs and the experimentally verified SFBSs implies that Lin et al. [18] applied very stringent filtering criteria in SCE detection, and thus there are certainly many multi-functional coding regions in the human genome that were not identified by them as SCEs.

Besides general statistical analyses, we have also examined a few important human proteins with different types of well-described overlapping functionalities. The correlation between overlapping DNA- and RNA-level regulatory sites and lack of local protein structure is clear in these cases, as well as the tendency of such multi-functional regions to reside outside well-folded domains. We further investigated a special case of triple functionality (Figure 5), in which splicing regulatory sites overlap with a longer region of dual protein coding and encode for structural/compositional biases in both protein isoforms.

It is important to emphasize that the coincidence between protein disorder and nucleotide-level functionality does not reveal the causative relationship between the two. There are two possible scenarios: (1) either the protein with an IDR existed first and the corresponding gene region could adopt an additional functionality due to the less stringent structural constraints of the encoded IDR, or (2) the other functionality existed first, which demanded reduced structural constraints in the overlapping protein. Obviously, none of these two scenarios might apply exclusively, since the ∼10000 examined multi-functional regions certainly provide examples for both. Human CBP and p300, for instance, seem to follow the first scenario. They are paralogues, showing a very high level of sequence conservation, but their detected SCEs are not similarly distributed along their chains (Figure 6). This implies that the overlapping regulatory functionalities represented by the detected SCEs appeared after the duplication of their ancestral gene i.e. the starting point of their evolutionary divergence. We have shown that these additional functionalities preferentially evolved at exon regions that could more easily accommodate them due to the lack of counteracting constraints in their encoded polypeptide chains.

In all, our results demonstrate that the level of complexity encoded by a genomic region of a given length is limited, and in case of multiple competing functions this limitation results in compromises. Since regulatory functions at DNA or RNA level are primarily fulfilled by short stretches of nucleotides, their information content cannot be reduced, which makes their sequences strictly conserved. Proteins, however, can be considered as longer functional elements, many of their residues are not crucial for their function and structural integrity, and are thus rather free to change. This is particularly true for regions of structural disorder and low sequence complexity, while globular domains are less flexible in this regard. In accord, we report here that genomic regions with multiple functionalities are more likely to overlap with protein regions of lower structural constraints, which suggests a trend towards the rational distribution of functional elements within the coding regions of genomes.

## Supporting Information

Figure S1 **Comparison of the SCE-containing subset of human proteins with the whole human canonical proteome from five aspects.** The lengths and the investigated structural properties of human SCE-containing proteins were compared to those of the human canonical proteome. The fraction of disordered residues (disorder content) was calculated based on predictions of the IUPred method and the fraction of residues in regions of low complexity was assigned according to SEG for each protein. The fraction of residues located in Pfam entities (domain content) was predicted by PfamScan, and the fraction of residues in secondary structure elements was calculated based on PSIPRED predictions. The sides of boxes show the 25th and the 75th percentile of the data, while the inner horizontal line indicates the median. The whiskers stand for the minimum and the maximum of the data. The two protein sets were compared by Mann-Whitney U test for each aspect and the corresponding p-values are provided above the boxes. Due to the multiplicity of comparisons performed on the two datasets, the significance thresholds were adjusted by Bonferroni correction (p = 0.01).
(TIF)

Figure S2 **Comparison of SCE-encoded protein segments with randomly selected segments of the SCE-containing subset of human proteins.** The structural properties of human SCE-encoded protein segments are compared to those of randomly selected segments from human SCE-containing proteins with the same length distribution. A) Fraction of disordered residues predicted by the IUPred method, B) fraction of residues in regions of SEG-assigned low sequence complexity, C) fraction of residues located in Pfam domains, and D) fraction of residues in secondary structure elements predicted by PSIPRED. The datasets are shown in the order of decreasing resolution (increasing window size) starting from the x axis, and each SCE dataset (dark grey) is followed by the equivalent random reference set (white). The sides of boxes correspond the 25th and the 75th percentile of the data, the vertical lines in the middle indicate the medians, while the small crosses stand for the means. The whiskers indicate the minimum and the maximum. The absence of boxes in case of the low sequence complexity data for the 9- and 15-codon resolution datasets indicate that the vast majority (>75%) of these smaller segments do not overlap with any low sequence complexity regions (only ∼9% of the human proteome is predicted as low complexity by the default SEG method). The corresponding SCE and reference segment sets were compared by Mann-Whitney U test for each aspect with the corresponding p-values provided next to the boxes.
(TIF)

Table S1 **Locations and predicted structural properties of protein regions overlapping the SCEs of the 9-codon resolution dataset.**
(XLSX)

Table S2 **Locations and predicted structural properties of protein regions overlapping the SCEs of the 15-codon resolution dataset.**
(XLSX)

Table S3 **Locations and predicted structural properties of protein regions overlapping the SCEs of the 30-codon resolution dataset.**
(XLSX)

Table S4 **Locations and predicted structural properties of segments randomly picked from human SCE-containing proteins with equivalent length distribution to the 9-codon resolution SCE dataset.**
(XLSX)

Table S5 **Locations and predicted structural properties of segments randomly picked from human SCE-containing proteins with equivalent length distribution to the 15-codon resolution SCE dataset.**
(XLSX)

Table S6 **Locations and predicted structural properties of segments randomly picked from human SCE-containing proteins with equivalent length distribution to the 30-codon resolution SCE dataset.**
(XLSX)

Table S7 **Structural properties of SCE-encoded protein regions compared to randomly selected segments from the SCE-containing subset of human proteins by Mann-Whitney U test.**
(DOCX)

Table S8 **Numbers of SCE-encoded protein segments assigned to the investigated structural properties compared to those of the randomly selected reference segments (Yates' chi-square test).**
(DOCX)

Table S9 **Comparison of the SCE-encoded protein segment datasets by Dunn's multiple comparison test.**
(DOCX)

Table S10 **Data on the filtered splicing factor binding site dataset.**
(XLSX)
