# Supplementary figures and images for: Synonymous Constraint Elements Show a Tendency to Encode Intrinsically Disordered Protein Segments
Source: PLoS Comput Biol. 2014 May 8;10(5):e1003607. doi: 10.1371/journal.pcbi.1003607 (PMC4014394; doi:10.1371/journal.pcbi.1003607)

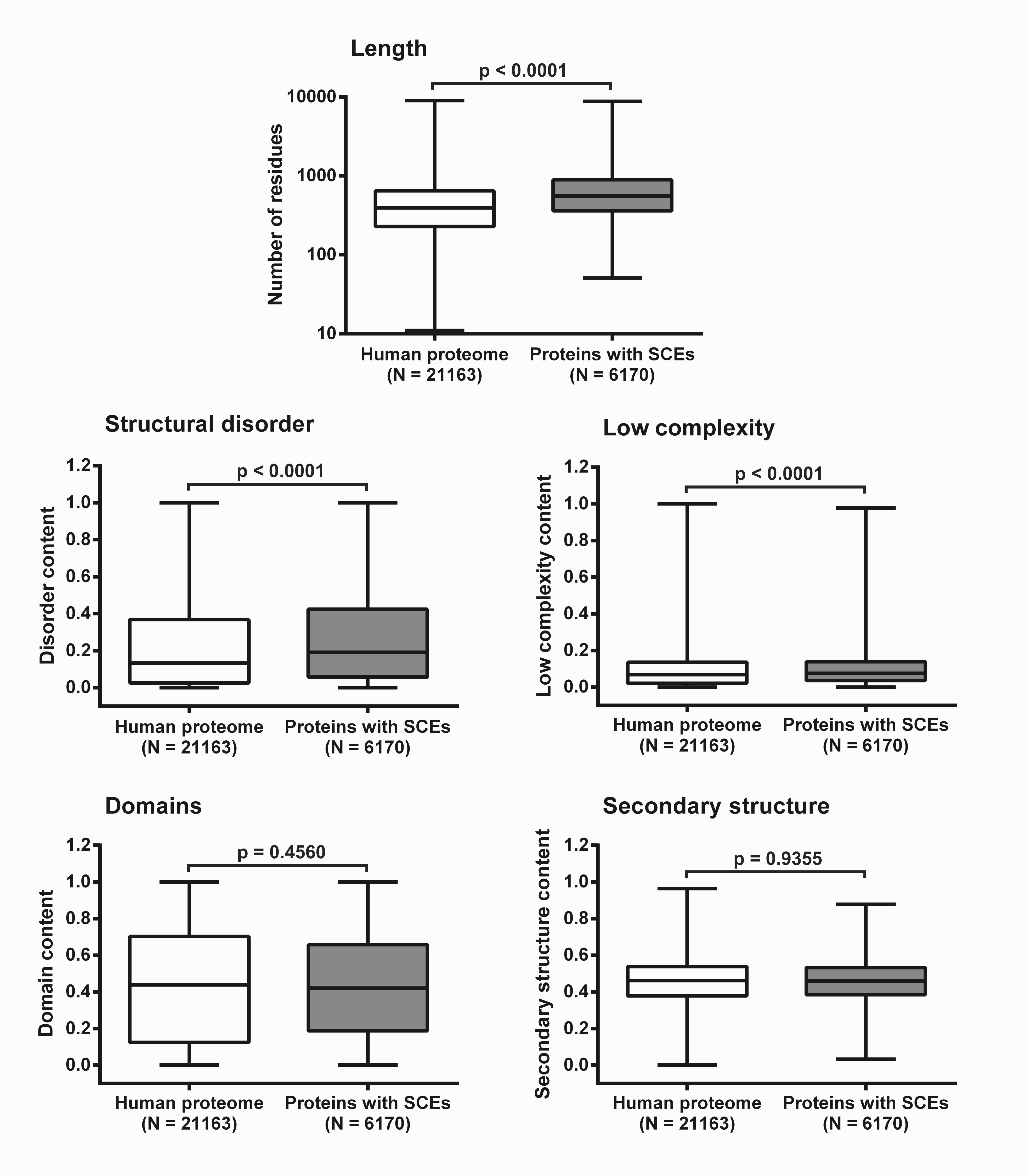

Supplement: Figure S1 — Comparison of the SCE-containing subset of human proteins with the whole human canonical proteome from five aspects. The lengths and the investigated structural properties of human SCE-containing proteins were compared to those of the human canonical proteome. The fraction of disordered residues (disorder content) was calculated based on predictions of the IUPred method and the fraction of residues in regions of low complexity was assigned according to SEG for each protein. The fraction of residues located in Pfam entities (domain content) was predicted by PfamScan, and the fraction of residues in secondary structure elements was calculated based on PSIPRED predictions. The sides of boxes show the 25th and the 75th percentile of the data, while the inner horizontal line indicates the median. The whiskers stand for the minimum and the maximum of the data. The two protein sets were compared by Mann-Whitney U test for each aspect and the corresponding p-values are provided above the boxes. Due to the multiplicity of comparisons performed on the two datasets, the significance thresholds were adjusted by Bonferroni correction (p = 0.01). (TIF) [file pcbi.1003607.s001.tif]

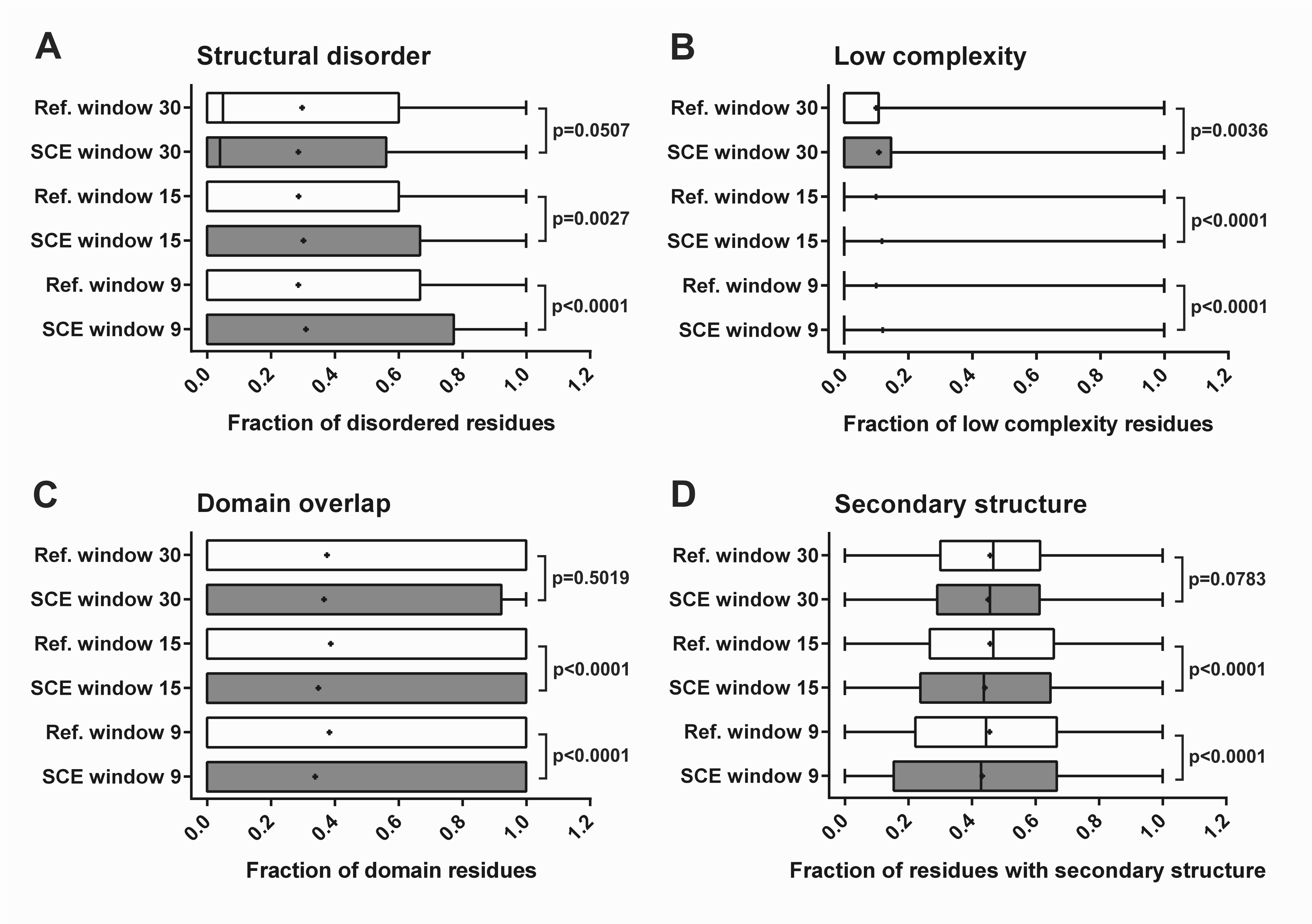

Supplement: Figure S2 — Comparison of SCE-encoded protein segments with randomly selected segments of the SCE-containing subset of human proteins. The structural properties of human SCE-encoded protein segments are compared to those of randomly selected segments from human SCE-containing proteins with the same length distribution. A) Fraction of disordered residues predicted by the IUPred method, B) fraction of residues in regions of SEG-assigned low sequence complexity, C) fraction of residues located in Pfam domains, and D) fraction of residues in secondary structure elements predicted by PSIPRED. The datasets are shown in the order of decreasing resolution (increasing window size) starting from the x axis, and each SCE dataset (dark grey) is followed by the equivalent random reference set (white). The sides of boxes correspond the 25th and the 75th percentile of the data, the vertical lines in the middle indicate the medians, while the small crosses stand for the means. The whiskers indicate the minimum and the maximum. The absence of boxes in case of the low sequence complexity data for the 9- and 15-codon resolution datasets indicate that the vast majority (>75%) of these smaller segments do not overlap with any low sequence complexity regions (only ∼9% of the human proteome is predicted as low complexity by the default SEG method). The corresponding SCE and reference segment sets were compared by Mann-Whitney U test for each aspect with the corresponding p-values provided next to the boxes. (TIF) [file pcbi.1003607.s002.tif]
