# Supplementary material for: Synonymous Constraint Elements Show a Tendency to Encode Intrinsically Disordered Protein Segments
Source: PLoS Comput Biol. 2014 May 8;10(5):e1003607. doi: 10.1371/journal.pcbi.1003607 (PMC4014394; doi:10.1371/journal.pcbi.1003607)
Supplement: Table S7 — Structural properties of SCE-encoded protein regions compared to randomly selected segments from the SCE-containing subset of human proteins by Mann-Whitney U test. (DOCX) [file pcbi.1003607.s009.docx]

**Table S7: Structural properties of SCE-encoded protein regions compared to randomly selected segments from the SCE-containing subset of human proteins by Mann-Whitney U test.**

|  | **Structural disorder content (IUPred)** | **Low-complexity content (SEG; window 12)** | **Secondary structure content (PSIPRED)** | **Fraction covered by Pfam Domains** |
| --- | --- | --- | --- | --- |
| **Dataset (Number of data (N))** | **Median^SCE^/median^random^, mean^SCE^/mean^random^, (Mann-Whitney U test p-value)** | **Median^SCE^/median^random^, mean^SCE^/mean^random^, (Mann-Whitney U test p-value)** | **Median^SCE^/median^random^, mean^SCE^/mean^random^, (Mann-Whitney U test p-value)** | **Median^SCE^/median^random^, mean^SCE^/mean^random^, (Mann-Whitney U test p-value)** |
| **SCE 9 (N = 11734)** | 0.000 / 0.000, 0.310 / 0.286, (p < 0.001) | 0.000 / 0.000, 0.120 / 0.100, (p < 0.001) | 0.429 / 0.444, 0.432 / 0.455, (p < 0.001) | 0.000 / 0.000, 0.338 / 0.383, (p < 0.001) |
| **SCE 15 (N = 10628)** | 0.000 / 0.000, 0.302 / 0.287, (p = 0.003) | 0.000 / 0.000, 0.118 / 0.099, (p < 0.001) | 0.438 / 0.467, 0.441 / 0.458, (p < 0.001) | 0.000 / 0.000, 0.349 / 0.387, (p < 0.001) |
| **SCE 30 (N = 8919)** | 0.040 / 0.050, 0.286 / 0.297, (p = 0.051) | 0.000 / 0.000, 0.108 / 0.099, (p = 0.004) | 0.457 / 0.467, 0.451 / 0.458, (p < 0.001) | 0.000 / 0.000, 0.366 / 0.375, (p = 0.502) |

Due to multiple comparisons performed on the SCE and corresponding reference datasets, we applied Bonferroni correction on the significance threshold (resulting in a threshold of p=0.0125).
