# Supplementary material for: Synonymous Constraint Elements Show a Tendency to Encode Intrinsically Disordered Protein Segments
Source: PLoS Comput Biol. 2014 May 8;10(5):e1003607. doi: 10.1371/journal.pcbi.1003607 (PMC4014394; doi:10.1371/journal.pcbi.1003607)
Supplement: Table S8 — Numbers of SCE-encoded protein segments assigned to the investigated structural properties compared to those of the randomly selected reference segments (Yates' chi-square test). (DOCX) [file pcbi.1003607.s010.docx]

**Table S8: Numbers of SCE-encoded protein segments assigned to the investigated structural properties compared to those of the randomly selected reference segments (Pearson’s chi^2^ test).**

|  | **Segments with at least 50% structural disorder (IUPred)** | **Segments with at least 50% low-complexity (SEG; window 12)** | **Segments with at least 50% secondary structure (PSIPRED)** | **Segments with at least 50% covered by Pfam domains (PfamScan)** |
| --- | --- | --- | --- | --- |
| **Dataset (number of data)** | **N^SCE (observed)^/N^ref (expected)^, Yeats corrected chi^2^, (chi^2^ test p-value)** | **N^SCE (observed)^/N^ref (expected)^, Yeats corrected chi^2^, (chi^2^ test p-value)** | **N^SCE (observed)^/N^ref (expected)^, Yeats corrected chi^2^, (chi^2^ test p-value)** | **N^SCE (observed)^/N^ref (expected)^, Yeats corrected chi^2^, (chi^2^ test p-value)** |
| **SCE 9 (N = 11734)** | 3617 / 3330, chi^2^ = 34.416 (p < 0.0001) | 1435 / 1169, chi^2^ = 66.972 (p < 0.0001) | 5104 / 5375, chi^2^ = 25.12, (p < 0.0001) | 4004 / 4526, chi^2^ = 97.819, (p < 0.0001) |
| **SCE 15 (N = 10628)** | 3140 / 2985, chi^2^ = 11.12 (p = 0.00085) | 1222 / 969, chi^2^ = 72.397 (p < 0.0001) | 4608 / 4855, chi^2^ = 23.041, (p < 0.0001) | 3754 / 4156, chi^2^ = 63.695, (p < 0.0001) |
| **SCE 30 (N = 8919)** | 2436 / 2532, chi^2^ = 5.03 (p = 0.0249) | 645 / 581, chi^2^ = 7.424 (p = 0.006436) | 3891 / 4082, chi^2^ = 16.393, (p < 0.0001) | 3336 / 3413, chi^2^ = 2.778, (p = 0.09557) |

Due to multiple comparisons performed on the SCE and corresponding reference datasets, we applied Bonferroni correction on the significance threshold (resulting in a threshold of p=0.0125).
