# Supplementary material for: Synonymous Constraint Elements Show a Tendency to Encode Intrinsically Disordered Protein Segments
Source: PLoS Comput Biol. 2014 May 8;10(5):e1003607. doi: 10.1371/journal.pcbi.1003607 (PMC4014394; doi:10.1371/journal.pcbi.1003607)
Supplement: Table S9 — Comparison of the SCE-encoded protein segment datasets by Dunn's multiple comparison test. (DOCX) [file pcbi.1003607.s011.docx]

**Table S9: Comparison of the SCE-encoded protein segment datasets by Dunn’s multiple comparison test for each structural property.**

|  | **Mean rank difference** | **P value** | **Significant?** |
| --- | --- | --- | --- |
| **Disorder content** |  |  |  |
| SCE 9 (N=11734) vs. SCE 15 (N = 10628) | -188.9 | 0.3549 | No |
| SCE 9 (N=11734) vs. SCE 30 (N = 8919) | -499.1 | 0.0003 | Yes |
| SCE 15 (N = 10628) vs. SCE 30 (N = 8919) | -310.1 | 0.0504 | No |
| **Low complexity content** | | | |
| SCE 9 (N=11734) vs. SCE 15 (N = 10628) | -516.3 | <0.0001 | Yes |
| SCE 9 (N=11734) vs. SCE 30 (N = 8919) | -1314 | <0.0001 | Yes |
| SCE 15 (N = 10628) vs. SCE 30 (N = 8919) | -797.6 | <0.0001 | Yes |
| **Domain residue content** | | | |
| SCE 9 (N=11734) vs. SCE 15 (N = 10628) | -274.0 | 0.0705 | No |
| SCE 9 (N=11734) vs. SCE 30 (N = 8919) | -743.5 | <0.0001 | Yes |
| SCE 15 (N = 10628) vs. SCE 30 (N = 8919) | -469.5 | 0.0009 | Yes |
| **Secondary structure content** | | | |
| SCE 9 (N=11734) vs. SCE 15 (N = 10628) | -451.9 | 0.0006 | Yes |
| SCE 9 (N=11734) vs. SCE 30 (N = 8919) | -896.7 | <0.0001 | Yes |
| SCE 15 (N = 10628) vs. SCE 30 (N = 8919) | -444.8 | 0.0018 | Yes |

Due to the multiplicity of structural properties compared between each pair of datasets, we applied Bonferroni correction on the significance thresholds (p = 0.0125).
